# Chemometric and learning-based multivariate models for quantifying a challenging quaternary mixture of bupropion, dextromethorphan, and their related impurities by UV-Vis spectrophotometry

**DOI:** 10.1186/s13065-026-01784-3

**Published:** 2026-04-12

**Authors:** Shrouk E. Algmaal, Shereen A. Boltia, Yasser S. El Saharty, Nermine S. Ghoniem

**Affiliations:** Pharmaceutical Analytical Chemistry Department, Faculty of Pharmacy, Cairo University, Kasr El-Ainy St., Cairo, 11562 Egypt

**Keywords:** Bupropion, Dextromethorphan, Multivariate methods, Greenness assessment

## Abstract

This study presents a robust, green, sustainable and time-efficient approach for the simultaneous determination of Bupropion HCl (BUP) and Dextromethorphan HBr (DEX) along with their related impurities 3-Chlorobenzoic acid and N, N-Dimethylaniline. Principal component regression (PCR) and partial least-squares (PLS), in addition to advanced chemometric models, namely multivariate curve resolution-alternating least squares (MCR-ALS), and artificial neural networks (ANN), are the four green smart multivariate spectrophotometric models that were proposed and validated. The suggested models were successful in examining the mixture of BUP and DEX in the presence of their impurities. Therefore, the suggested analytical methods can be applied to pharmaceutical formulation analysis without the need for a separation step. The proposed strategy offers a novel analytical platform for quality control laboratories to manage complex formulations involving interfering substances. To further ensure greenness and sustainability of the proposed approach, several assessment tools were applied, including the Modified National Environmental Methods Index (NEMI), Eco-Scale, the Analytical GREEnness (AGREE) metric, the Hexagon algorithm, the Green Analytical Procedure Index (GAPI), the Modified GAPI (MoGAPI), the Blue Applicability Grade Index (BAGI), White Analytical Chemistry (WAC), and the Click Analytical Chemistry Index (CACI). Many traditional analytical techniques pose undesirable dangers to the environment and the analyst, such as using hazardous solvents. This gave analysts the incentive to use green methodologies that take into account the use of safe chemicals, the production of the least amount of trash, substantial time savings, and enhanced analyst safety.

**Supplementary Information:**

The online version contains supplementary material available at 10.1186/s13065-026-01784-3.

## Introduction

**Fig. 1:**
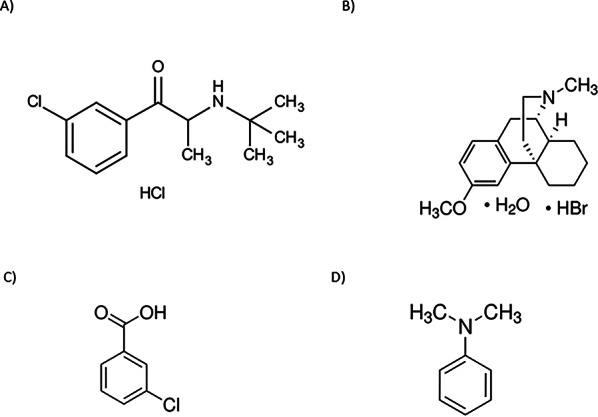
Chemical structures of (**A**) Bupropion hydrochloride (BUP), (**B**) Dextromethorphan hydrobromide (DEX), (**C**) 3-chlorobenzoic acid (CBA), and (**D**) N.N-dimethylaniline (DMA)

 Atypical antidepressant and smoking cessation medication bupropion hydrochloride (BUP, Fig. 1A) [2-(tert-butylamino)-1-(3-chlorophenyl) propan-1-one hydrochloride] is frequently combined with dextromethorphan hydrobromide (DEX, Fig. 1B) [(9α,13α,14α)-3-Methoxy-17-methylmorphinan hydrobromide], a common antitussive that may have antidepressant synergy. Accurate quality control in dosage forms is becoming more and more important as both medications are being studied in combination therapies. Their drug formulations could include hazardous process-related contaminants that need close monitoring, like 3-Chlorobenzoic acid (3-CBA, Fig. 1C), a bupropion metabolite that is physiologically inactive and a known contaminant in pharmaceutical raw materials and final products with a limit of not more than 0.2% [1, 2] and N, N-dimethylaniline (DMA, Fig. 1D), a process-related impurity formed during the manufacture of dextromethorphan, which is highly toxic and carcinogenic and has an acceptable limit of not more than 0.001% [1].

Examination of the literature implies that BUP and DEX have been measured using various analytical techniques including HPTLC [2], HPLC [3, 4], UV-spectrophotometry [5, 6], and voltammetry [7], with the latter applied specifically to BUP in the presence of DEX. On the other hand, the literature survey revealed that no analytical methods have been reported for the concurrent determination of BUP and DEX together with 3-CBA and DMA, which are the United State Pharmacopeia USP official impurities of BUP and DEX, respectively. The majority of the published analytical methods employed traditional approaches that generated a lot of waste and required a lot of organic solvents. Furthermore, these analytical methods primarily presented environmental harm and health risks to the analyst. Despite the growing interest in chemometric-assisted spectroscopic methods for pharmaceutical analysis, it is important to acknowledge their intrinsic limitations, particularly in quantitative trace-level determination. International regulatory guidelines, such as those issued by the International Council for Harmonisation (ICH), require validated analytical methods with reliably estimated figures of merit, including sensitivity, limits of detection (LOD), and limits of quantification (LOQ). Although several theoretical and practical approaches have been proposed since the 1990s to estimate these parameters in multivariate calibration [8–11], most notably through multivariate detection limit concepts and net analytical signal theory, a universally harmonized framework fully aligned with current regulatory validation requirements has not yet been established.

Furthermore, chemometric models such as partial least squares (PLS), artificial neural networks (ANN), and multivariate curve resolution–alternating least squares (MCR-ALS) operate without prior separation or preconcentration steps. While this feature enables efficient quantification of major components in complex and highly overlapping systems, it may limit applicability in trace-level analysis. In such cases, matrix-associated interferences and the absence of analyte enrichment can increase estimation uncertainty and reduce analytical reliability. Consequently, chromatographic techniques remain the reference methods for the determination of low-level or potentially toxic pharmaceutical impurities due to their superior selectivity and robustness, even when more elaborate sample preparation is required. Within this framework, chemometric spectroscopic approaches should be viewed as complementary, green, and cost-effective alternatives rather than replacements for separation-based methods.

Chemometrics employs mathematical and computational tools to extract meaningful analytical information from noisy signals, such as those obtained via UV-Vis spectroscopy. The resolution of the analytes and impurities using conventional UV is challenging due to the strong spectral overlap between them. Chemometric models using principal component regression (PCR), partial least squares (PLS), multivariate curve resolution-alternating least squares (MCR-ALS) and learning model based on artificial neural networks (ANN) have been used in this study. Most widely used multivariate calibration models in chemometrics are PCR and PLS. These models make it possible to minimize noise, reduce signal interference, and resolve overlapped spectra [12].

In recent years, more resolution-based advanced models, like MCR-ALS, has been used for multivariate calibration in which number of constraints were attempted in order to reduce the number of potential solutions and enhance the quantification of the concentration profiles of the chemicals [13]. Non–linear-based learning model, like ANN is more applied, owing to the greater power and progressive capabilities of computers. It uses a variety of approaches to mimic a number of the cognitive functions of the human brain. It is thought to be better than other classical linear multivariate models (PCR, PLS), including classical non-linear-based learning model (ANN) [14].

The use of green chemistry has recently been recognized by international scientific communities as having numerous positive social and economic impacts in addition to making the planet more environmentally friendly and more sustainable. Analytical chemists employ green chemistry operational perspective to develop or choose efficient evaluation approaches, generate minimal waste, and employ chemicals that are safe for the environment and people. One example of a green analytical chemistry view point that can reduce waste and the amount of solvents used is to analyze only the smallest number of samples at the smallest sample size. Consequently, a variety of strategies and recommendations for choosing a solvent have been developed [15, 16]. Most of these standards are somewhat intricate spreadsheets that rank the consequences on safety, health, and the environment along with recommendations for replacing toxic solvents. However, choosing the best solvent was difficult because of the sheer volume of parameters displayed in these spreadsheets. To help with solvent selection depend on their physicochemical properties, Safety-Health-Environmental (SHE) impact, and the regulatory considerations, an interactive, open-access online technology was recently introduced [17, 18]. Developing, refining, and validating environmentally friendly multivariate calibration models (PLS, PCR, MCR-ALS, ANN) and for the simultaneous measurement of BUP and DEX in the presence of their impurities in bulk and pharmaceutical formulations are the aim of this work. Finally, to guarantee the greenness of the developed study, the following strategies were used: Modified National Environmental Methods Index (NEMI), Eco-scale, The Analytical GREEnness (AGREE), Hexagon algorithm, Green Analytical Procedure Index (GAPI), Modified Green Analytical Procedure Index (MoGAPI), The blue applicability grade index (BAGI), White Analytical Chemistry (WAC), and The Click Analytical Chemistry Index (CACI).

## Experimental

### Instrument and software

A dual beam UV-visible spectrophotometer with two identical quartz cells that is connected to PC is the Shimadzu UV-1605 PC (Japan).The absorption spectra were processed using the UV-PC personal spectroscopy software version (3.7). Multivariate calibrations were calculated using Matlab^®^ R2018a (The Mathworks, Natick, MA, USA).

The solvent option was made using the solvent selection tool developed by the American Chemical Society Green Chemistry Institute Pharmaceutical Roundtable (ACS GCIPR) available online at https://acsgcipr.org/tools/solvent-tool/. The neural network toolboxes free software available at http://www.mcrals.info and MCR-ALS were set up and utilized in Matlab^®^.

### Materials and reagents

Pure standards of BUP, DEX, 3-CBA, and DMA were purchased from Sigma-Aldrich (USA), with purities of 99.84 ± 1.46% for BUP and 100.34 ± 1.43% for DEX, as verified by their respective official HPLC USP and potentiometric BP methods, respectively [1, 19]. AUVELITY^®^ extended-release tablets (BN: 81968-045-30) made by Axsome Therapeutics, Ind., New York, USA. It contains 105 mg BUP and 45 mg dextromethorphan per tablet. Butan-2-ol (Sigma Aldrich, Darmstadt, Germany).

### Standard solutions

Stock standard solutions of BUP, DEX, 3-CBA, and N, N-DMA (1.00 mg. mL^− 1^, each) were made by weighing 100.00 mg of each drug into four different 100 mL volumetric flasks, followed by the addition of butan-2-ol. The solutions were sonicated until they dissolved, and then butan-2-ol was added to complete them off. From their respective stock standard solutions, working standard solutions of BUP, DEX, 3-CBA, and N, N-DMA were made with a concentration of 100.00 µg mL^− 1^.

### Procedures

#### Making a solvent decision

The ACS Green Chemistry Institute Pharmaceutical Roundtable (ACS GCIPR) solvent selection tool, which is based on principal component analysis (PCA) of 70 physicochemical parameters for 272 solvents, was employed to guide solvent selection. In the present study, screening criteria focused on solvent physical properties, ICH classification, and solvent health, safety, and environmental (SHE) impact. Application of the lowest SHE rating narrowed the initial list to ten candidate solvents, which was subsequently reduced to three ICH Class 3 solvents. Based on physicochemical suitability and cost-effectiveness, butan-2-ol was selected as the optimal green solvent for the experimental work.

#### Spectral characteristics and absorption spectra

The scanning range for the absorption spectra of BUP, DEX, 3-CBA, and DMA was 200.0–400.0 nm. Spectrum data points, which ranged from 224.0 to 330.0 nm, were chosen and sent to MATLAB^®^ for additional data processing.

#### Construction of calibration and validation sets

A five-level, four-factor calibration design was used to create the calibration and validation sets [20].The calibration set was created utilizing 17 mixtures with varying concentrations of BUP, DEX, 3- CBA, and DMA and 8 samples as the validation set in the ranges of 5.00–25.00, 8.00–16.00, 2.00–10.00, and 2.00–10.00 µg mL^− 1^, respectively. In 10 mL volumetric flasks, different aliquots of their working solutions were combined and diluted with the appropriate quantity of butan-2-ol. The resultant solutions were scanned in the range of 200.0–400.0 nm. The spectral data in the 224.0–330.0 nm range were imported into MATLAB for data manipulation and calibration model creation using 1 nm intervals. 107 experimental points were used. Before developing the PCR, PLS, MCR-ALS, ANN and models, the spectroscopic data were mean-centered. Latent variable (LV) numbers were optimized using leave-one-out cross-validation for the PCR and PLS models. The ideal LVs for the PCR and PLS models were 6 and 5, respectively that matched the least significant calibration error were ideal. A rough estimate of the number of participating elements in the system under investigation is the first stage in the MCR-ALS model. These preliminary findings are then refined by an iterative approach utilizing the alternative least square constraint. Since applied constraints are the main optimization parameter in the MCR-ALS approach, extra constraints can be implemented during the optimization phase to direct the outcome toward a chemical meaning. When convergence is reached, the iteration process is over. To gain suitable parameters with the fewest number of iterations, we employed horizontal unimodality for both concentration and spectral profiles in the study we presented. The wavelength range of 224.0–330.0 nm was the one that over which the spectra of the generated solutions were recorded and scanned at 1-nm intervals. The calculations used 107 experimental points. For further data processing, the data points from the spectra were transferred into Matlab^®^. In this work, a feed-forward artificial neural network was developed and trained using the Levenberg–Marquardt backpropagation algorithm.In the input layer, 107 neurons were used to denote the number of spectral data points used, whereas four neurons were employed in the output layer, which denotes the number of components to be assessed (DMA, 3-CBA, DEX, and BUP). To achieve the optimal network design, several elements must be modified, including the learning rate, the number of epochs, and the number of nodes in the hidden layer. It was discovered that utilizing a purelin-purelin transfer function produced the best results with ten hidden neurons. A validation set including eight samples was used to assess the prediction potential of the calibration models of PCR, PLS, MCR-ALS, and ANN.

### Assay of pharmaceutical dosage form

Ten AUVELITY^®^ tablets were carefully weighed, finely ground using a hand mortar, and homogenized. A quantity of powder corresponding to 105 mg of BUP and 45 mg of DEX was put to a conical flask which contains 30 mL of butan-2-ol.

To guarantee full extraction, the mixture was sonicated for half an hour. The final solution was quantitatively transferred into a 100 mL volumetric flask and filled with butan-2-ol. Whatman^®^ grade 1 filter paper was then used to filter the previous solution. From this solution, 1.0 mL was transferred into a 50 mL volumetric flask and then completed volume with butan-2-ol .The ultimate concentration of the previous solution was stated to be 21.00 µg mL^− 1^ BUP and 9.00 µg mL^− 1^ DEX. The proposed chemometric models were applied to analyze the pharmaceutical preparation using the previously described analytical methods, after which the levels of BUP and DEX were determined.

### Determination of BUP and DEX by official methods

Bupropion was determined according to the procedure described in the United States Pharmacopeia [1]. The method employs HPLC using a C18 column and a mobile phase composed of methanol, tetrahydrofuran, and phosphate buffer (pH 7.0) in the ratio of 39:11:50 (v/v/v). The analysis was performed at a flow rate of 1.1 mL min⁻¹ with UV detection at 250 nm.

Dextromethorphan was determined according to the British Pharmacopoeia [19]. Briefly, 0.300 g of the substance was dissolved in a mixture of 5.0 mL of 0.01 M hydrochloric acid and 20 mL of ethanol (96%). The resulting solution was titrated with 0.1 M sodium hydroxide, and the endpoint was determined potentiometrically. The volume of sodium hydroxide consumed between the two inflection points was recorded. Each 1.0 mL of 0.1 M sodium hydroxide is equivalent to 35.23 mg of C₁₈H₂₆BrNO.

## Results and discussion

**Fig. 2:**
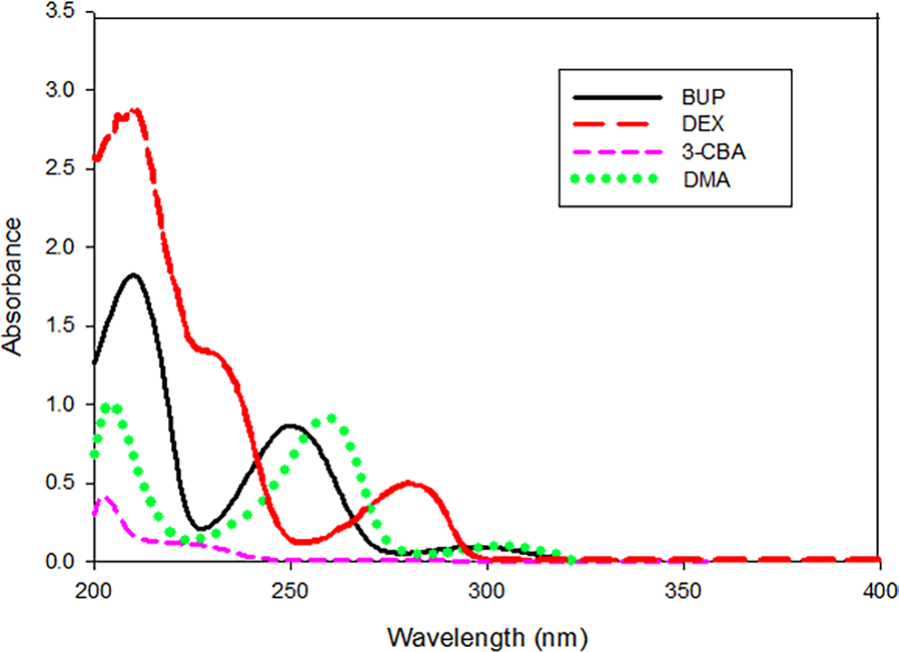
Absorption spectra of 20.00 µg mL^− 1^ BUP (**—**), 20.00 µg mL^− 1^ DEX (_ _), 5.00 µg mL^− 1^ 3-CBA (- - - -), and 3.00 µg mL^− 1^ DMA (**…….**), using butan-2-ol as solvent

Multivariate analysis allows for the effective resolution of severely overlapping spectroscopic data by simultaneously utilizing multiple spectral variables across different wavelengths. In contrast to univariate analysis, which relies on a single absorbance value at a selected wavelength, this approach provides enhanced analytical specificity and sensitivity [21]. The significant spectral overlap prevents the quantification of the combined medications in Auvelity^®^ tablets using a univariate spectrophotometric approach (Fig. 2). Therefore, to achieve their successive quantification, chemometric models (PCR, PLS, MCR-ALS), and learning model (ANN) were constructed. Only a few analytical methods for figuring out BUP and DEX in pharmaceutical formulations have been documented; however, no chemometric models for figuring out BUP and DEX mixtures in the presence of their official impurities have been presented. Consequently, it was crucial to create a precise analytical method for concurrently identifying the active substances and contaminants that could be present in pharmaceutical dosage forms. Concurrent advancements in chemometrics, analytical instrumentation, and computational capabilities have facilitated the development of effective tools for the visualization and interpretation of complex chemical data. Multivariate calibration models like MCR-ALS resolve extensively overlapping spectra by simultaneously utilizing information from multiple spectral wavelengths, providing improved accuracy and precision compared to single-wavelength methods. Chemometric models enable rapid prediction of analyte concentrations through multifactorial analysis of spectra obtained from unknown samples. As a result, multivariate calibration models are commonly applied in quality control laboratories for impurity profiling [22]. Chemometrics may also be used to create metabolic profiles and has a number of other biomedical uses [23].

### Making a solvent selection

Several solvent scales have been developed to numerically classify, characterize, and rank solvents. Frequently univariate, these scales attempted to assess a single characteristic of the solvents [24–26]. 272 solvents were initially shown by the ACS GCIPR online tool based on a number of parameters [17, 27]. First, the solvents with the lowest ranking for SHE attributes (from 1 to 3) were chosen as the 10 greenest solvents. Water, 1-methoxy-2-propanol, perfluorodecalin, triethylene glycol, 2-methyl butanol, methyl isobutyl carbinol, 3-methylbutan-1-ol, butan-2-ol, and pentan-1-ol are some of these solvents. Then, by choosing the solvent class that the ICH categorization indicated, our search was restricted to three solvents.

**Fig. 3:**
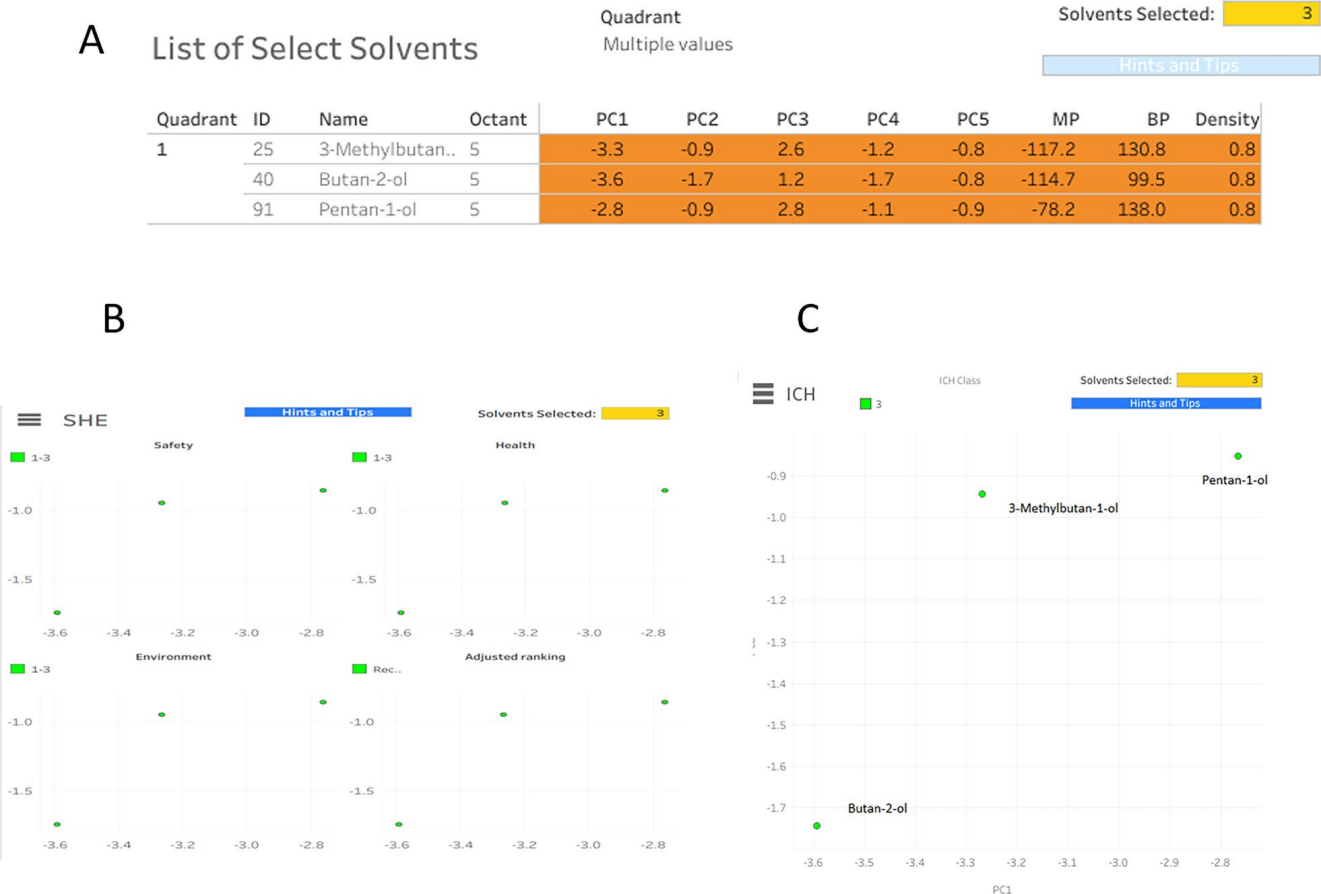
(**A**–**C**) Results of the solvent selection presented in the solvent map in the (ACS GCIPR) online tool

The options were narrowed down to pentan-1-ol, butan-2-ol, and 3-methylbutan-1-ol (Fig. 3A). Because they satisfy the green criteria of both the SHE (Fig. 3B) and ICH (Fig. 3C) recommendations, these two steps yielded the three greenest solvents that the program could provide. Due to the intermediate polarity of butan-2-ol enables adequate solubilization of both the polar and moderately nonpolar components of the studied drugs. It is also the ideal environmentally friendly option for our investigative experimental circumstances. Finally, depending on its physical qualities and cost-effectiveness, we determined that butan-2-ol was the optimal green solvent for our preliminary experimental setup.

### Calibration and validation set

Twenty-five combinations were used to validate and calibrate the proposed models Table 1. The absorbance data of the samples was selected from 224.0 to 320.0 nm.

Since all the components indicated absorbance characteristics within this range, this range was selected. Wavelengths shorter than 224.0 nm were eliminated to eliminate the influence of noise in the calibration matrix. Additionally, wavelengths longer than 320.0 nm were not included since they were thought to provide less useful absorbance information.

### Principal component regression (PCR), partial least squares (PLS) models

In chemometrics, PCR and PLS have garnered a lot of interest for multicomponent analysis. Identifying the best approach is still a difficult task [28, 29] .When there is little knowledge available about the components, these models work especially well. PCR produces elements that improve data interpretability without accounting for the response variable. On the other hand, PLS incorporates the response variable into its analysis and often generates models that fit the response variable with fewer components [30]. In order to produce a more reliable model, PLS eliminates interference from absorbance and concentration data.

**Fig. 4:**
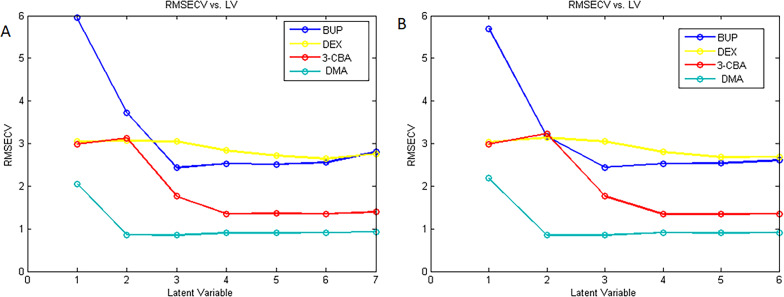
RMSEC plot of the cross-validation results of the calibration set as a function of the number of latent variables used to construct (**a**) PCR calibration and (**b**) PLS calibration

A cross-validation leave-one-out that excluded one sample at a time was used to determine the optimal number of variables. Six LVs were found to be the most effective for PCR, and five LVs for PLS in our analysis (Fig. 4A and B).

### Multivariate curve resolution-alternating least squares (MCR-ALS)

Spectroscopic techniques are widely used in quantitative analysis because spectral measurements are easy to perform and interpret. However, standard spectrophotometric techniques rely on a limited range of wavelengths, often excluding important information needed to distinguish components with overlapping spectra. On the other hand, the benefit of using the entire set of spectral data points is provided by multivariate techniques. By examining the correlations between each variable and every other variable, MCR builds a regression model [31, 32]. The Beer-Lambert law is expanded to the multi-wavelength domain by this model [33]. The spectroscopic data matrix in MCR is broken down into pure spectral-profile and concentration-profile matrices before a residual’s matrix is computed. Using MCR-ALS, the concentrations of the components under study are iteratively estimated from their spectral profiles. To obtain meaningful concentration and pure spectral profiles, the ALS optimization applies several constraints, including unimodality, closure, equality, and non-negativity. An additional advanced constraint was introduced into the MCR-ALS framework to obtain pure resolved profiles in arbitrary units without reference quantitative information [13, 14]. By adding a correlation constraint, which adds an inner calibration step to the MCR-ALS model, this problem is further resolved [14].

**Fig. 5:**
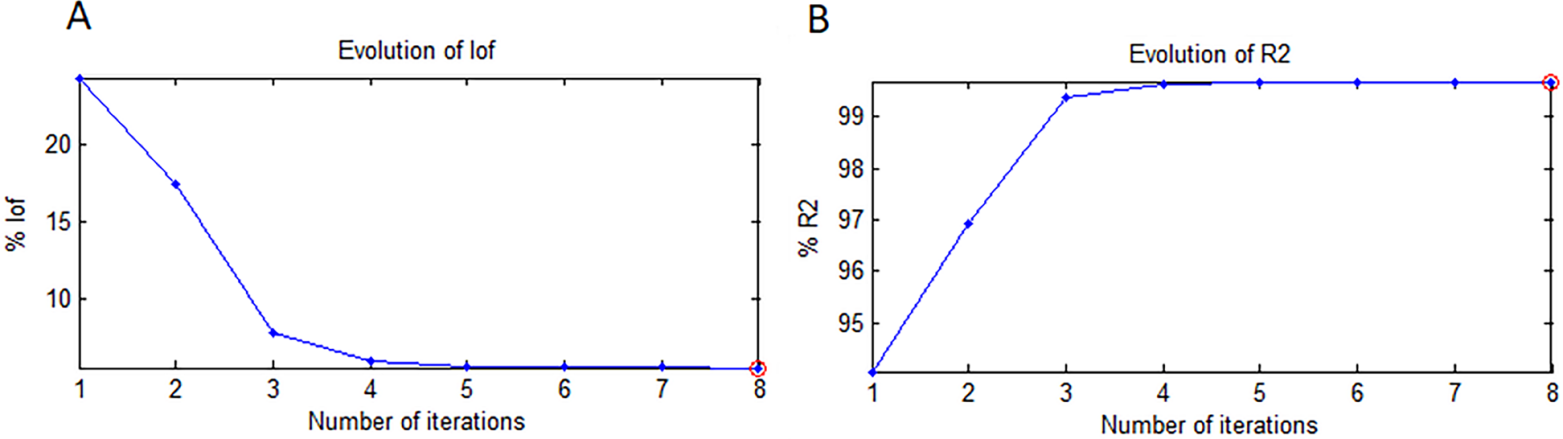
(**A**) Percentage lack of fit and (**B**) variance percentage of MCR-ALS model

The order of components may alter during ALS optimization due to rotational ambiguity without impacting the data matrix [34]. Convergence was typically achieved at 0.1% [13], and in our case, convergence was reached after five iterations. The estimated figures of merit for the optimization process variance percentage (R²) and lack of fit were 99.6865% and 1.5193%, respectively, which were considered acceptable and confirmed the robustness of the developed MCR-ALS model (Fig. 5). Because the MCR-ALS model can estimate the spectral profiles of the drugs under study, it also makes qualitative interpretation possible. Using a one-by-one test sample approach, the corresponding spectral and concentration profiles were recovered during ALS optimization in order to determine the concentrations of the components in the validation set [14] .

**Fig. 6:**
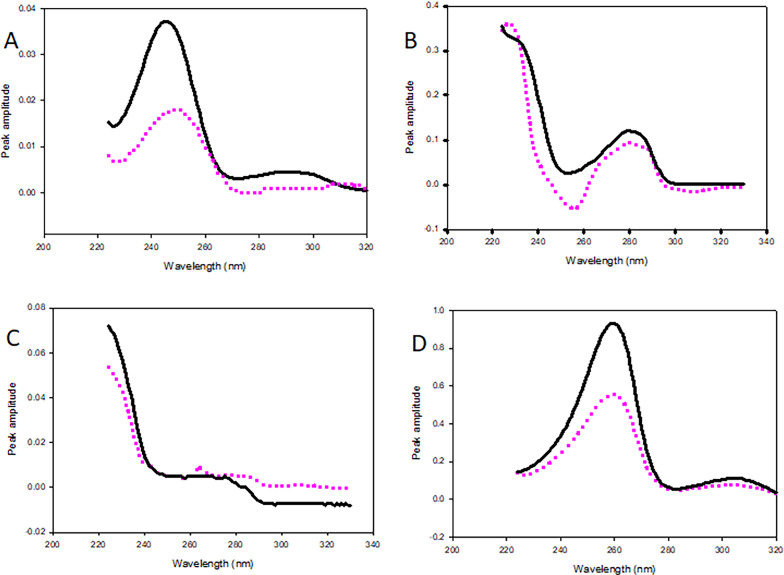
Pure spectra ( **___**) and extracted spectra by MCR-ALS (……… ) for the four components of (**A**) BUP, (**B**) DEX, (**C**) 3-CBA and (**D**) DMA

The optimization process was terminated once the predefined convergence threshold was met after the minimum number of iterations. Table 2 presents predicted concentrations. The calculated spectra were nearly identical to the original spectra (Fig. 6), confirming that, in addition to its quantitative capability, the MCR-ALS model provides valuable qualitative component resolution.

### Artificial neural networks (ANN)

**Fig. 7:**
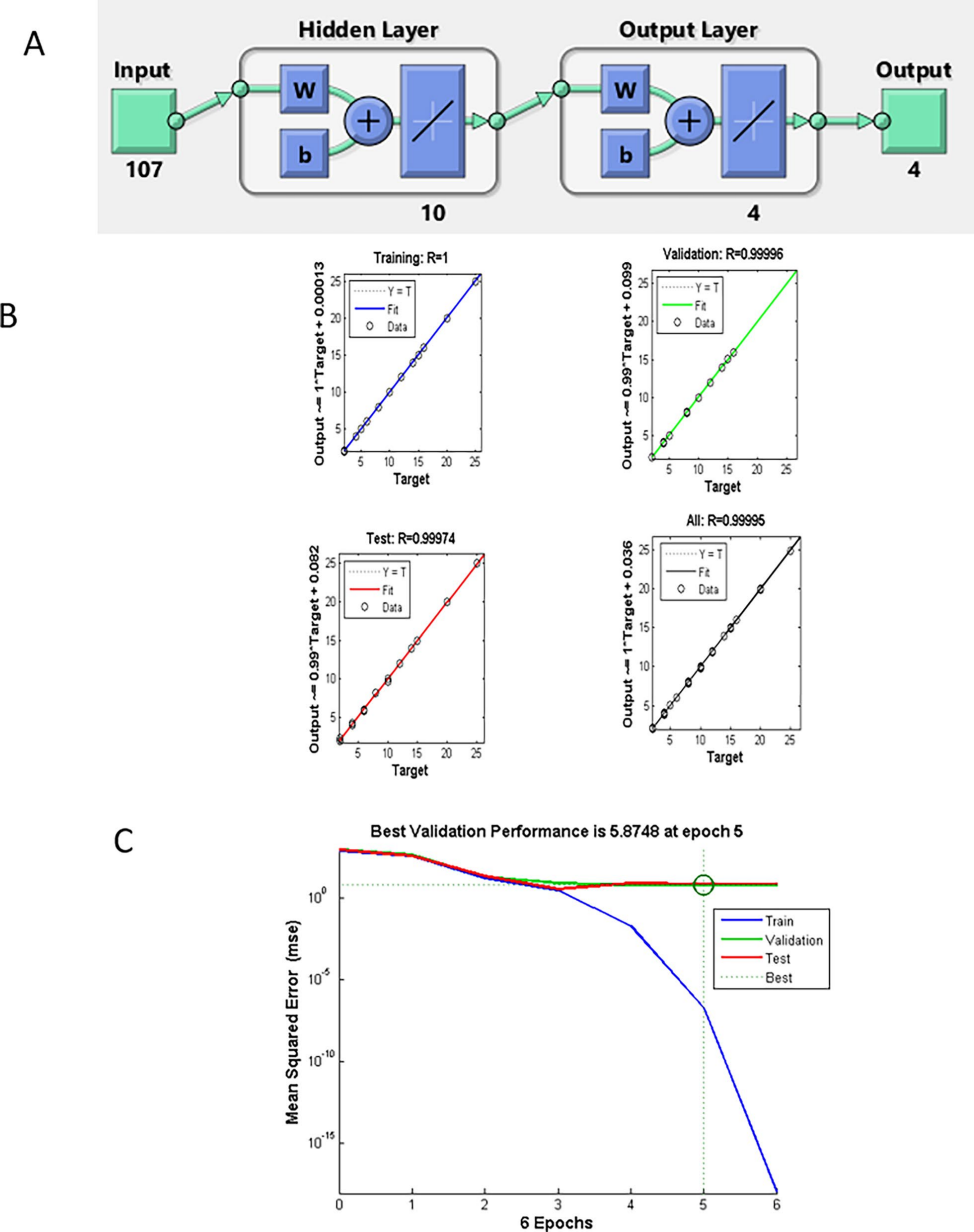
(**A**) The structure of the ANN model with inputs, outputs, hidden layers and the number of neurons in the hidden layer (**B**) Prediction for the training, test, and validation diagrams of the ANN model (**C**) Best validation performance for the prediction of the ANN model

**Fig. 8:**
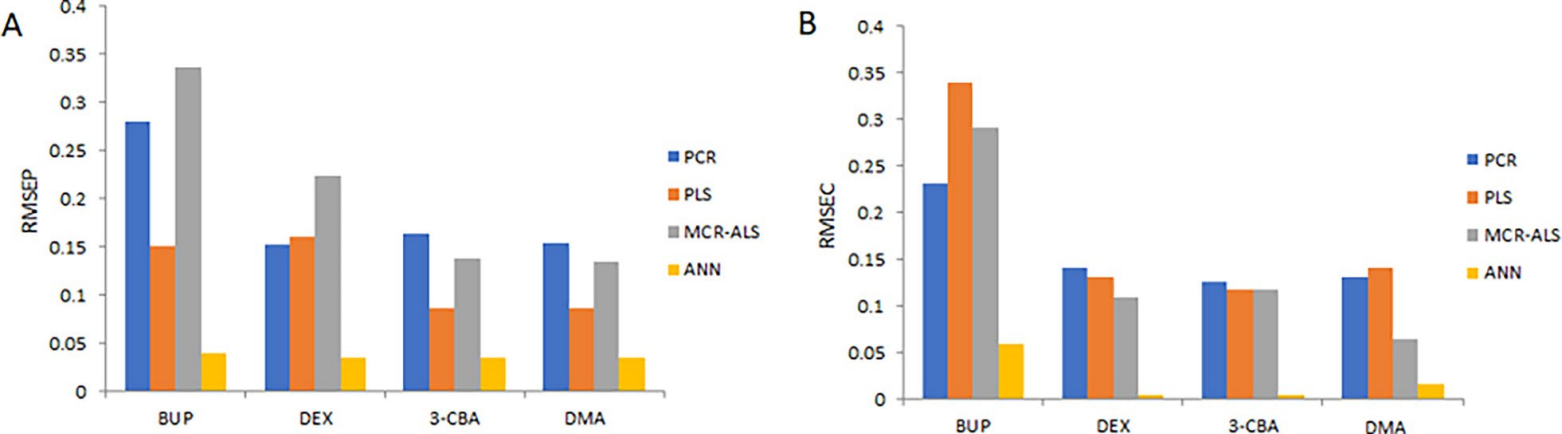
The calculated (**A**) RMSEC for each component achieved by the proposed calibration models and (**B**) RMSEP calculated by the corresponding validation model

It is a non-linear learning model that mimics the function of the biological nervous system by using a large number of interconnected nodes, or artificial neurons, to identify relationships between inputs and outputs. When modeling non-linear interactions among variables, ANN outperforms other commonly used multivariate models such as PCR and PLS [35], because these linear multivariate models (PCR, PLS, etc.) were conceived to deal with alinear relationship between instrumental analytical signals (e.g., absorbance) and target sample properties (e.g., concentration). A feed-forward model was the type of ANN used in this study. The input layer consisted of 107 neurons, corresponding to the number of spectroscopic data points, while the output layer contained four neurons, matching the number of compounds to be quantified in each sample. Using a purelin–purelin transfer function, ten neurons were found to be the optimal number for the hidden layer. Figure 7A illustrates the architecture of the ANN model, including the input, hidden, and output layers and the number of neurons in each. The schematic predictions for the training, test, and validation sets for the selected layers and neurons are shown in Fig. 7B, with no noticeable differences observed between the test and validation patterns. The fully trained model’s mean squared error (MSE) performance is shown in Fig. 7C. ANN across epochs showed a gradual decrease in MSE after epoch 5. The predictive performance of the chemometric models (PCR, PLS, MCR-ALS) and learning model (ANN) was evaluated using spectra from the validation set. For each component, relative standard deviations (RSDs) and average recoveries were computed (Table 3), demonstrating positive outcomes. Regression and validation parameters for pure samples of BUP, DEX, 3-CBA, and DMA are displayed in Table 4. Using the developed models, the concentrations of the four components in the validation set mixtures were determined, and the root mean square error of prediction (RMSEP), recovery percentage, and RSD were calculated, yielding satisfactory outcomes (Table 3). The root mean square error of calibration (RMSEC) and RMSEP for the calibration and validation models of each component were also computed. The elliptical joint confidence region (EJCR) test was used as a statistical test [36, 37]. The EJCR test was implemented to provide a statistically objective comparison between MCR-ALS and ANN, clearly demonstrating the superior precision and accuracy of ANN, as evidenced by smaller confidence regions and closer proximity to the ideal point. Elliptical joint confidence region (EJCR) analysis at the 95% confidence level was applied to objectively compare the predictive performances of the MCR-ALS and ANN models. As shown in Fig.S1 the EJCRs were constructed from the regression of predicted versus nominal concentrations, where the ideal unbiased model is represented by the point (slope = 1, intercept = 0). The ANN confidence regions are tightly clustered around the ideal point, whereas the MCR-ALS ellipses show greater dispersion and displacement, confirming the superior predictive accuracy, higher precision, lower systematic bias, and robustness of ANN across the studied concentration ranges. Column charts illustrating these RMSEC and RMSEP values are presented in Fig. 8. All of the findings supported the notion that ANN is the preferred model for quantifying drug mixture under study. Furthermore, the pure spectral profiles of the four components can only be distinguished by the MCR-ALS model. It might therefore be used for both qualitative and quantitative analysis.

### Assay of pharmaceutical formulation

Both BUP and DEX were evaluated in their dosage form, Auvelity^®^ tablets, using the developed chemometric models. The accurate quantification of the four drugs in the pharmaceutical product was confirmed by the satisfactory recovery percentages, with standard deviations below 2% (Table 5).

### Statistical analysis

There was no significant difference in accuracy and precision between the official methods [1, 19] and the chemometric approaches devised in this investigation, according to the statistical analysis shown in Table 6.

For the repeatability study, three representative validation mixtures (covering low, medium, and high concentration levels) were each prepared and analyzed in triplicate within a single day. The spectra were processed using the finalized models (PCR, PLS, MCR-ALS, ANN), and the predicted concentrations were used to calculate the relative standard deviation (%RSD).

For the intermediate precision study, the same validation samples were freshly prepared and analyzed on three different days. The overall %RSD across all days was calculated. The obtained %RSD values for all analytes were consistently below 2% for repeatability and below 2.5% for intermediate precision across all four models, confirming the excellent precision and robustness of the proposed methods for routine analysis. Detailed results are provided in Supplementary Material, Table S1.

In addition, statistical assessment of accuracy (trueness) for BUP and DEX was performed in accordance with IUPAC recommendations using Student’s *t*-test. The mean recoveries obtained from all models (PCR, PLS, MCR-ALS, and ANN) were statistically compared with the reference value of 100%. The corresponding experimental *t* values (*t*ₑₓₚ) were calculated and evaluated against the critical *t* values at the 95%, 97.5%, and 99% confidence levels (*t*₍crit,95%₎ = 2.365, *t*₍crit,97.5%₎ = 2.960, *t*₍crit,99%₎ = 3.499; df = 7).

As presented in Table S2, all calculated *t*ₑₓₚ values for BUP and DEX were lower than the corresponding critical thresholds, confirming the absence of statistically significant systematic bias. These findings demonstrate that the proposed analytical models provide accurate and reliable results. Importantly, this analysis highlights that numerical proximity of recoveries to 100% alone is insufficient to establish trueness, and that formal hypothesis testing is essential for statistically sound validation.

Accordingly, the proposed chemometric approaches for BUP and DEX comply with IUPAC-consistent validation principles [38, 39].

**Table 1:** Concentrations of BUP, DEX, and their impurities 3-CBA, DMA in the calibration and validation sets for the multivariate calibration models

Mixture no.	Conc. (µg mL^− 1^ )			
	BUP	DEX	3-CBA	DMA
1^a^	15.00	12.00	6.00	6.00
2	15.00	8.00	4.00	2.00
3^a^	5.00	10.00	2.00	10.00
4	10.00	8.00	10.00	10.00
5	5.00	16.00	10.00	6.00
6^a^	25.00	16.00	6.00	4.00
7^a^	25.00	12.00	4.00	10.00
8	15.00	10.00	10.00	4.00
9	10.00	16.00	4.00	8.00
10^a^	25.00	10.00	8.00	8.00
11	10.00	14.00	8.00	6.00
12	20.00	14.00	6.00	10.00
13^a^	20.00	12.00	10.00	8.00
14	15.00	16.00	8.00	10.00
15	25.00	14.00	10.00	2.00
16	20.00	16.00	2.00	2.00
17	25.00	8.00	2.00	6.00
18	5.00	8.00	6.00	8.00
19	5.00	12.00	8.00	2.00
20	15.00	14.00	2.00	8.00
21^a^	20.00	8.00	8.00	4.00
22	5.00	14.00	4.00	4.00
23^a^	20.00	10.00	4.00	6.00
24	10.00	10.00	6.00	2.00
25	10.00	12.00	2.00	4.00

^a^Represent the validation set mixtures

**Table 2:** Prediction concentrations of validation set samples using the proposed chemometric models

Sample no.	Nominal concentration (µg mL^−1^)				PCR				PLS			
					Predicted concentration				Predicted concentration			
	BUP	DEX	3-CBA	DMA	BUP	DEX	3-CBA	DMA	BUP	DEX	3-CBA	DMA
1	15.00	12.00	6.00	6.00	15.23	12.16	5.93	5.94	14.98	11.89	6.11	5.94
3	5.00	10.00	2.00	10.00	5.03	10.04	2.00	9.73	5.06	10.16	2.01	9.83
6	25.00	16.00	6.00	4.00	25.37	16.30	5.83	4.05	25.09	15.61	6.10	3.94
7	25.00	12.00	4.00	10.00	24.6	11.89	4.06	9.94	25.00	11.89	4.06	9.94
10	25.00	10.00	8.00	8.00	24.57	10.07	7.95	8.14	24.87	10.09	7.82	8.02
13	20.00	12.00	10.00	8.00	19.82	12.14	9.61	8.09	20.33	12.05	9.98	7.89
21	20.00	8.00	8.00	4.00	19.84	7.95	8.06	3.92	19.86	8.01	7.94	3.92
23	20.00	10.00	4.00	6.00	20.15	9.82	4.14	5.97	20.15	9.99	3.99	5.97

Sample No.	Nominal concentration (µg mL^−1^)				MCR-ALS				ANN			
					Predicted concentration				Predicted concentration			
	BUP	DEX	3-CBA	DMA	BUP	DEX	3-CBA	DMA	BUP	DEX	3-CBA	DMA
1	15.00	12.00	6.00	6.00	15.29	13.73^a^	5.82	5.99	14.83	12.00	6.00	6.00
3	5.00	10.00	2.00	10.00	4.88	10.27	2.02	10.2	4.90	10.01	2.00	10.00
6	25.00	16.00	6.00	4.00	27.15	15.82	6.04	4.11	25.00	15.99	5.90	4.00
7	25.00	12.00	4.00	10.00	25.40	12.17	3.89	10.04	25.00	12.01	4.00	10.00
10	25.00	10.00	8.00	8.00	25.63	10.44^a^	8.11	8.16	24.90	10.00	8.00	8.00
13	20.00	12.00	10.00	8.00	20.09	12.19	9.74	8.23	19.66	12.30	10.00	8.00
21	20.00	8.00	8.00	4.00	20.34	8.26	8.10	4.11	20.00	8.00	8.00	4.00
23	20.00	10.00	4.00	6.00	21.32^a^	10.25	4.11	5.99	20.00	9.96	4.00	5.90

^a^ Rejected values according to Q rejection rule

**Table 3:** Results of determination of BUP, DEX and their impurities in the validation set using the proposed models

Sample No.	Nominal concentration (µg mL^− 1^)					PCR				PLS			
	BUP	DEX	3-CBA		DMA	BUP	DEX	3-CBA	DMA	BUP	DEX	3-CBA	DMA
1	15.00	12.00	6.00		6.00	101.53	101.34	98.9	99.08	99.87	99.12	101.85	99.08
3	5.00	10.00	2.00		10.00	100.55	100.41	100.01	97.29	101.26	101.62	100.59	98.26
6	25.00	16.00	6.00		4.00	101.50	101.84	97.12	101.22	100.36	97.59	101.66	98.38
7	25.00	12.00	4.00		10.00	98.38	99.11	101.56	99.41	99.98	99.1	101.6	99.41
10	25.00	10.00	8.00		8.00	98.26	100.70	99.40	101.69	99.5	100.89	97.81	100.19
13	20.00	12.00	10.00		8.00	99.59	101.19	96.10	101.16	101.66	100.41	99.8	98.67
21	20.00	8.00	8.00		4.00	99.18	99.36	100.77	97.89	99.29	100.16	99.31	97.99
23	20.00	10.00	4.00		6.00	100.76	98.18	103.40	99.47	100.77	99.86	99.74	99.55
					**Mean**	**99.97**	**100.27**	**99.66**	**99.65**	**100.33**	**99.84**	**100.29**	**98.94**
					**RSD%**	**1.31**	**1.26**	**2.36**	**1.61**	**0.83**	**1.25**	**1.40**	**0.76**
					**RMSEP**	**0.2803**	**0.1529**	**0.1639**	**0.1556**	**0.1506**	**0.1615**	**0.0872**	**0.0863**

Sample No.	Nominal concentration (µg mL^− 1^)					MCR-ALS				ANN			
	BUP	DEX	3-CBA	DMA		BUP	DEX	3-CBA	DMA	BUP	DEX	3-CBA	DMA
1	15.00	12.00	6.00	6.00		101.96	114.44^a^	96.96	99.84	98.84	100.00	100.00	100.00
3	5.00	10.00	2.00	10.00		99.67	102.71	101.01	101.96	97.99	100.09	99.99	100.00
6	25.00	16.00	6.00	4.00		100.61	98.87	100.65	102.84	100.00	99.94	98.33	100.00
7	25.00	12.00	4.00	10.00		101.59	101.43	97.20	100.36	100.00	100.04	100.00	99.99
10	25.00	10.00	8.00	8.00		100.11	104.44^a^	101.42	101.98	99.60	100.02	100.00	100.00
13	20.00	12.00	10.00	8.00		100.43	101.55	97.36	102.92	98.30	102.50	100.00	100.00
21	20.00	8.00	8.00	4.00		101.69	103.28	101.31	102.74	100.00	100.00	100.00	99.99
23	20.00	10.00	4.00	6.00		106.62^a^	102.46	102.71	99.86	100.00	99.64	100.00	98.33
				**Mean**		**100.87**	**101.72**	**99.83**	**101.56**	**99.34**	**100.28**	**99.79**	**99.79**
				**RSD%**		**0.87**	**1.67**	**2.12**	**1.22**	**0.85**	**0.91**	**0.59**	**0.59**
				**RMSEP**		**0.3374**	**0.2234**	**0.1383**	**0.1342**	**0.1439**	**0.1069**	**0.0353**	**0.0353**

^a^ Rejected values according to Q rejection rule

**Table 4:** Performance parameters of the calibration set calculated for each proposed model

Parameter	PCR model				PLS model			
	BUP	DEX	3-CBA	DMA	BUP	DEX	3-CBA	DMA
Slope	0.992	1.0202	1.0021	0.9892	1.0165	0.9848	0.9989	0.9921
Intercept	0.0202	0.2462	0.0347	0.0392	0.0438	0.2335	0.0407	-0.0284
Correlation coefficient (r)	0.9994	0.9991	0.9991	0.9991	0.9995	0.9992	0.9993	0.9991
RMSEC	0.2307	0.1408	0.1264	0.1307	0.3396	0.1306	0.1166	0.1401

Parameter	MCR-ALS model				ANN model			
	BUP	DEX	3-CBA	DMA	BUP	DEX	3-CBA	DMA
Slope	1	1	1	1	0.9958	0.9889	0.9823	0.9968
Intercept	−4.84 × 10^− 16^	−9.10 × 10^− 16^	3.62 × 10^− 16^	1.14 × 10^− 16^	0.113	0.1077	0.0966	0.0164
Correlation coefficient (r)	0.9990	0.9994	0.9993	0.9997	0.9993	0.9998	0.9998	0.9991
RMSEC	0.2908	0.1085	0.1179	0.0637	0.0588	0.0035	0.0031	0.0161

**Table 5:** Quantitative determination of BUP and DEX in the dosage form by the proposed chemometric models

Pharmaceutical formulation	Drugs	PCR	PLS	MCR-ALS	ANN
		Recovery % ± RSD%*			
Auvelity^®^ tablet	BUP	100.25 ± 1.18	100.57 ± 0.67	100.01 ± 0.37	100.01 ± 0.39
	DEX	100.82 ± 1.57	100.34 ± 1.24	100.03 ± 1.04	100.03 ± 0.25

*Average of three determinations

**Table 6:** Statistical comparison of the results obtained by the proposed chemometric models and the official methods for the determination of BUP, and DEX in their pure powdered form

Parameter Drugs	PCR		PLS		MCR-ALS		ANN		Official methods ^a, b^	
	BUP	DEX	BUP	DEX	BUP	DEX	BUP	DEX	BUP ^**a**^	DEX ^**b**^
Mean	99.97	100.27	100.33	99.84	101.87	101.72	99.34	100.28	99.84	100.34
S.D.	1.31	1.27	0.84	1.24	0.88	1.67	0.84	0.91	1.459	1.429
n	8	8	8	8	7	6	8	8	3	3
Variance	1.716	1.613	0.706	1.538	0.774	2.789	0.708	0.824	2.129	2.041
F value ^c^	1.874 (4.737)	1.906 (4.737)	4.549 (4.737)	1.977 (4.737)	4.117 (5.143)	1.255 (5.786)	4.511 (4.737)	3.873 (4.737)	–	–
Student’s t-test^c^	0.133 (2.262)	0.078 (2.262)	0.652 (2.262)	0.533 (2.262)	1.265 (2.306)	1.204 (2.365)	0.657 (2.262)	0.077 (2.262)	–	–

^a^BUP is determined using an HPLC method [1]

^b^DEX is determined potentiometrically [19]

^c^The values in parentheses represent the corresponding tabulated *t* and *F* values at a significant level of *p* = 0.05

**Table 7:** Comparison between proposed and reported method in terms of greenness, blueness and whiteness

Parameter	Chemometric method	Reported HPTLC method
Environmental Methods Index (NEMI)		
The Hexagon algorithm		
Green Analytical Procedure Index (GAPI)		
Modified Green Analytical Procedure Index (MoGAPI)		
The blue applicability grade index (BAGI)		
White and The Click Analytical Chemistry Index (CACI)		
Analytical Chemistry (WAC)		
White and The Click Analytical Chemistry Index (CACI)		

### Assurance of methodology greenness

The environmental, health, and safety impact of analytical methods can be evaluated using various greenness metrics [2, 40, 41]. The greenness of analytical methods was evaluated according to the 12 principles of Green Analytical Chemistry (GAC) using several complementary tools [42]. The NEMI pictogram, including its 2009 update, indicated predominantly green zones, reflecting reduced solvent consumption and waste compared to the HPTLC method [43, 44]. The Eco-Scale score of the proposed method was 90 versus 76 for HPTLC, demonstrating improved sustainability [45]. The Eco-Scale scores methods by subtracting penalties for energy, solvents, exposure, and waste from 100, providing a simple, quantitative measure of greenness [46]. The Eco-Scale evaluation of our proposed experimental setup yielded a score of 90, which is considered a good score according to its criteria [45]. The AGREE assessment yielded a score of 0.79, compared to 0.66 for HPTLC, primarily due to the use of safer solvents [47].

The Hexagon approach showed lower penalty scores for cost, toxicity, waste, and analytical performance [48]. The Hexagon tool assigns penalties (0–4) across six criteria, and the proposed method showed lower scores than HPTLC, reflecting better performance, safety, and cost-effectiveness [48].

GAPI was used to assess environmental impact, using a five-section color-coded pentagram to indicate low, medium, and high risk across sample collection, reagent toxicity, energy use, and waste [7], The proposed method showed more green and fewer red zones than HPTLC. The modified GAPI (MoGAPI) pictogram, with color-coded segments, further confirmed its superior greenness and provides web-based labeling for easy interpretation [49]. The tool provides an overall score for easy comparison. The proposed method, with mostly green and few yellow/red segments, scored 80 versus 72 for HPTLC, confirming its lower environmental impact.

The Blue Applicability Grade Index (BAGI) was determined following the methodology introduced by Manousi et al.) [50] using the BAGI online platform (bagi-index.anvil.app). The BAGI index demonstrated superior operational applicability and efficiency, scoring 90.

The WAC metric indicated higher overall sustainability [51]. Compared to the reported approach, which had a score of 92.6, the suggested method received a score of 94.3.

The practicality and cost-effectiveness of the approach was assessed using Click Analytical Chemistry Index (CACI) program (bit.ly/CACI2025).CACI index confirmed excellent practicality and cost-effectiveness.

This method enables analysts to determine the advantages and disadvantages of a technique [52]. Table 7 presents a comparison of sustainability indices of proposed chemometric method versus published HPTLC method [2] .

In summary, the green color that predominates in the modified NEMI, GAPI and MoGAPI graphs, the high score of AGREE, Eco-scale, WAC, and CACI and the hexagon algorithm’s low score, and all guaranteed that the suggested analytical method would adhere to the GAC principles and, consequently, be sustainable. The chosen solvent and the analysis method, which called for fewer samples and hence generated less waste with less chemical exposure for the analyst, are responsible for the results obtained.

## Conclusion

The continuous developments in chemometrics have made it possible to separate and analyze the chemical data in addition to univariate analysis. The integration of chemometric strategies with UV-Vis spectroscopy represents a powerful evolution in analytical methodology, enabling the extraction of meaningful information from noisy second-order signals with minimal resource demands. Unlike traditional chromatography, which relies on extensive solvent use, large sample volumes, and optimized separations, multi-way calibration such as MCR-ALS facilitates accurate resolution of overlapping profiles even at low chromatographic efficiency, enhances selectivity, sensitivity, and sustainability by reducing optimization needs and environmental impact, aligning seamlessly with green analytical chemistry (GAC) principles. Our study demonstrates these benefits through successful application to complex UV-Vis matrices, underscoring chemometrics’ transformative role in bridging conventional limitations with efficient, eco-friendly quantification. The suggested chemometric models have demonstrated exceptional sensitivity and dependability in measuring BUP, DEX, 3-CBA, and DMA concurrently in a quick, easy, and reliable manner. Early in the creation process, the models’ environmental friendliness was considered. In terms of accuracy and precision, there were no statistically significant variations between the official and established ones. Therefore, the suggested green multivariate models offer a useful and eco-friendly substitute for the conventional analysis of measuring BUP, DEX, 3-CBA, and DMA in pharmaceutical formulations and bulk powder.

## Supplementary Information

Below is the link to the electronic supplementary material.

Supplementary Material 1.

## Data Availability

The datasets used and/or analysed during the current study are available from the corresponding author on reasonable request.
