# Supplementary material for: Chemometric and learning-based multivariate models for quantifying a challenging quaternary mixture of bupropion, dextromethorphan, and their related impurities by UV-Vis spectrophotometry
Source: BMC Chem. 2026 Apr 12;20(1):87. doi: 10.1186/s13065-026-01784-3 (PMC13085295; doi:10.1186/s13065-026-01784-3)
Supplement: Supplementary file 1 — Supplementary Material 1. [file 13065_2026_1784_MOESM1_ESM.docx]

**Table S1**. Repeatability and intermediate precision of BUP, DEX and their impurities in the validation set using the proposed models.

| **Precision (RSD%)** |  | **BUP** | **DEX** | **3-CBA** | **DMA** |
| --- | --- | --- | --- | --- | --- |
| **Repeatability** | PCR | 0.546 | 0.719 | 1.478 | 1.195 |
|  | PLS | 0.698 | 0.272 | 0.272 | 0.446 |
|  | MCR-ALS | 0.703 | 0.703 | 0.306 | 0.370 |
|  | ANN | 0.088 | 0.074 | 0.305 | 0.039 |
| **Intermediate precision** | PCR | 1.844 | 1.366 | 2.237 | 1.986 |
|  | PLS | 1.188 | 1.328 | 0.669 | 0.791 |
|  | MCR-ALS | 0.978 | 0.835 | 0.498 | 0.498 |
|  | ANN | 0.403 | 0.430 | 0.576 | 0.417 |

**Table S2.** Statistical evaluation of accuracy (trueness) according to IUPAC recommendation

| **PCR** | **Analyte** | **Mean (%)** | **RSD %** | **tₑₓₚ** |
| --- | --- | --- | --- | --- |
|  | BUP | 99.97 | 1.31 | 0.070 |
|  | DEX | 100.27 | 1.26 | 0.602 |
| **PLS** | BUP | 100.33 | 0.83 | 1.120 |
|  | DEX | 99.84 | 1.25 | 0.359 |
| **MCR-ALS** | BUP | 100.87 | 0.87 | 2.80 |
|  | DEX | 101.72 | 1.67 | 2.86 |
| **ANN** | BUP | 99.34 | 0.85 | 2.210 |
|  | DEX | 100.28 | 0.91 | 0.872 |

**Critical value t_(0.05, 7)_= 2.365**

**Critical value t_(0.025, 7)_ = 2.998**

**Critical value t_(0.01, 7)_ = 3.499**


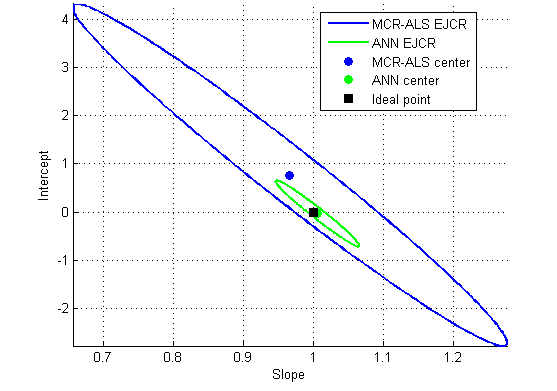


Fig.S1. Elliptical joint confidence region (EJCR) plots at the 95% confidence level for obtained using MCR-ALS and ANN models. The ellipses represent the joint confidence regions of slope and intercept derived from predicted versus nominal concentration regressions. The ideal point (slope = 1, intercept = 0) corresponds to an unbiased model.
